# Abutting Objects Warp the Three-Dimensional Curvature of Modally Completing Surfaces

**DOI:** 10.1177/2041669520903554

**Published:** 2020-04-09

**Authors:** Peter U. Tse

**Affiliations:** Department of Psychological and Brain Sciences, Dartmouth College, Hanover, New Hampshire, United States

**Keywords:** 3D perception, binocular vision, contours/surfaces, shape, surfaces/materials

## Abstract

Binocular disparity can give rise to the perception of open surfaces or closed curved surfaces (volumes) that appear to vary smoothly across discrete depths. Here I build on my recent papers by providing examples where modally completing surfaces not only fill in from one depth layer’s visible contours to another layer’s visible contours within virtual contours in an analog manner, but where modally completing surface curvature is altered by the interpolation of an abutting object perceived to be connected to or embedded within that modally completing surface. Seemingly minor changes in such an abutting object can flip the interpretation of distal regions, for example, turning a distant *edge* (where a surface ends) into *rim* (where a surface bends to occlude itself) or turning an open surface into a closed one. In general, the interpolated modal surface appears to deform, warp, or bend in three-dimensions to accommodate the abutting object. These demonstrations cannot be easily explained by existing models of visual processing or modal completion and drive home the implausibility of localistic accounts of modal or amodal completion that are based, for example, solely on extending contours in space until they meet behind an occluder or in front of “pacmen.” These demonstrations place new constraints on the holistic surface and volume generation processes that construct our experience of a three-dimensional world of surfaces and objects under normal viewing conditions.

The central point made by Kanizsa’s (1955) famous triangle demonstration was that surfaces are interpolated on the basis of both contour cues and cues about visual occlusion. In this singular iconic drawing, he made clear that there is modal completion of the triangle in front of the pacmen, which must be induced by the pacmen, and, simultaneously, amodal completion of the disks that are occluded by the modally completed triangle, which, by occluding those disks leaves only pacmen visible. Following in his tradition, 2 years ago (Tse, 2017a, 2017b), I introduced a new class of binocular surface and volume completion demonstrations that placed further constraints on the surface and volume generation processes that construct our three-dimensional (3D) world under normal viewing conditions. Those demonstrations raised issues that cannot be easily explained by existing models of visual processing.

The traditional examples of amodal and modal completion introduced by Kanizsa (1955) or Carman and Welch (1992), involved open surfaces (i.e., those that do not close on themselves, into a volume) that complete modally in front of pacmen-like inducers. The visible and illusorily completed contours of these unclosed Kanizsa-style figures correspond to an edge in the world; An edge occurs where a surface is taken to just end, like the side of a piece of paper. My new (Tse, 2017a, 2017b) demonstrations involved visible and illusorily completed contours that are generally not taken to arise from edge in the world but instead from portions of “rim.” Rim, unlike edge, occurs where the line of sight just grazes, tangentially, a smooth, or differentiable surface. There is no unique tangent at an edge. But there is a unique tangent at the rim, which, for everywhere differentiable surfaces, lies along the line of sight. The rim forms a curve (or a set of disjoint 3D curves) on the surface of a 3D object that together comprise the dividing border between visible and self-occluded parts of the object. Because there are two eyes displaced in space, each eye has a different set of rim segments that lie on the surface of the object; contour differences seen by the two eyes, if taken to arise from rim differences, permit the interpolation of a smooth surface between those rim segments locally, and around the front and back of the object to distant rim segments, non-locally, in 3D. Because illusory surfaces are taken to continue in front of, behind, and beyond the visible or illusory contour arising from the rim of the modally completing surface, they close into a volume that encloses space. Thus, curved 3D surfaces are interpolated by the visual system to vary smoothly across depths in binocularly fused images, even when only two (or more) discrete binocular disparities are defined between corresponding elements of the inducing image contours. These illusory surfaces are generated in the 3D space inferred to lie between the two (or more) disparity-defined depths, and only arise in uniform regions where there are no disparity cues that could define depth upon binocular fusion, whether crossed or uncrossed. Surfaces are filled-in from one depth layer’s visible contour fragments to another layer’s visible contour fragments within virtual contours that are themselves interpolated on the basis of good contour continuation in 3D (i.e., the extent to which contours connect modally or amodally as a function of the degree of interpolated coalignment in 3D space; Tse, 1999a, 1999b) and other contour-based cues. Such interpolated 3D surfaces may pass through visible contours along the line of sight or at some other angle. The interpolated surface solution is influenced by nonlocal cues: When there are two or more surface-propagating contour segments, they can merge and possess a depth and perceived surface curvature that is consistent with all visible contour segments despite the absence of local disparity cues in regions far from any inducing contours. Indeed, because surfaces are assumed to close smoothly, there are cases where the interpolated curved closed surfaces appear to lie closer or farther than the nearest or farthest visible depth, respectively, implied by binocular disparity cues at visible contours. The present work builds on this past work and demonstrates the ways in which an object, interpreted to lie on or within a modally completing surface, can bend or warp that surface in 3D in a manner that cannot be explained solely in terms of localistic contour interactions such as emphasized in the contour relatability account of Kellman et al. (Ghose et al., 2014; Kellman, Garrigan, & Shipley, 2005; Kellman et al., 2007; Kellman, Garrigan, Shipley, Yin, et al., 2005; Kellman & Shipley, 1991; Kellman et al., 1998; Shipley & Kellman, 1992; Yin et al., 1997; but see also Anderson, 2007a, 2007b; Anderson et al., 2002; Boselie & Wouterlood, 1992; Singh & Hoffman, 1999; Singh et al., 1999; Tse, 1999a, 1999b) that built on the idea of boundary completion operations modeled by Grossberg and Mingolla (1985a, 1985b) and Grossberg (1994).

## Demonstrations

**Figure 1.**
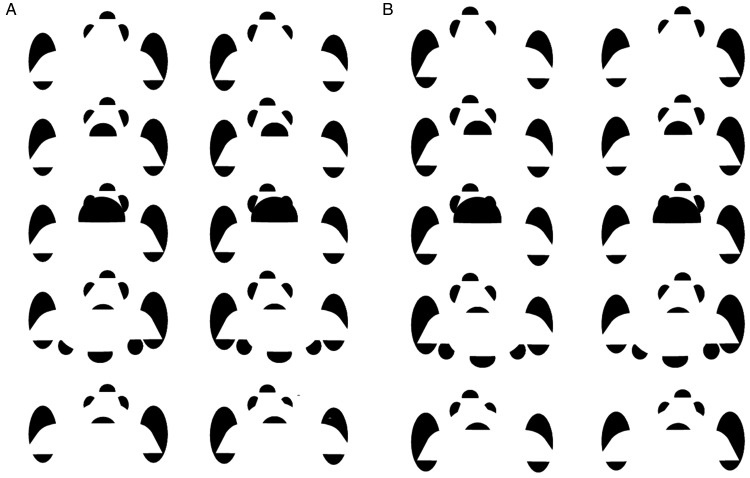
Five versions of a curvy “children’s slide” from the top (a) to bottom row (e) vary depending on the placement of embedded “balls”: a crossed-disparity version (A), an uncrossed-disparity version (B), an animated crossed-disparity version (Movie 1), and an uncrossed-disparity version (Movie 2).*Note:* Figure 1a refers to the top row, Figure 1b refers to the next row down.

We can begin with an open curved surface, such as the curvy “children’s slide” depicted in Figure 1a. (Note that both crossed- and uncrossed-disparity versions of each figure and animation are provided here. The reader should observe the crossed or uncrossed version of each figure, depending on their personal preferred mode of binocular viewing.) Adding a “ball” to the children’s slide’s surface as in Figure 1b would seem to cause the ball to bend down around the presumed base of the ball, which is taken to be occluded by the slide itself. In this case, the “side edges” of the slide are not pressed down, but the surface of the slide around the base of the ball must be pressed down, if this is indeed taken to be a ball, warping the shape of the slide in 3D that is presumed to support its weight, as if it were a rubber sheet being pressed down by a heavy ball. Whereas the front of the slide is entirely visible in Figure 1a, this is no longer the case in Figure 1b, where the front of the slide now bends and self-occludes. (An animated version of this is shown in Movies 1 and 2.) If we make the ball bigger as in Figure 1c, this might even push the illusory contours, corresponding to “edges” of the slide, down as well. In this case, it is remarkable that the upper portion of the slide seems to link up with the lower edges of the slide because the to-be-completed contours are occluded by the presence of the ball, eliminating any contour relatability in the image. Adding a “basin” to the slide, as in Figure 1d, leads to surface completion of the slide, now from the front first to the basin, and only then to the far end of the slide. Moreover, the ball in Figure 1d is now taken to reside, semioccluded in the basin. Adding slight modal occlusions of the lower portion of the rear pacmen, as in Figure 1e, creates the impression that the slide deforms into a “ring” around the ball, rather like a nest, while maintaining the smooth differentiability of the modally completing surface.

**Movie 1.** (click to play). Crossed Disparity.

**Movie 2.** (click to play). Uncrossed Disparity.

**Figure 2.**
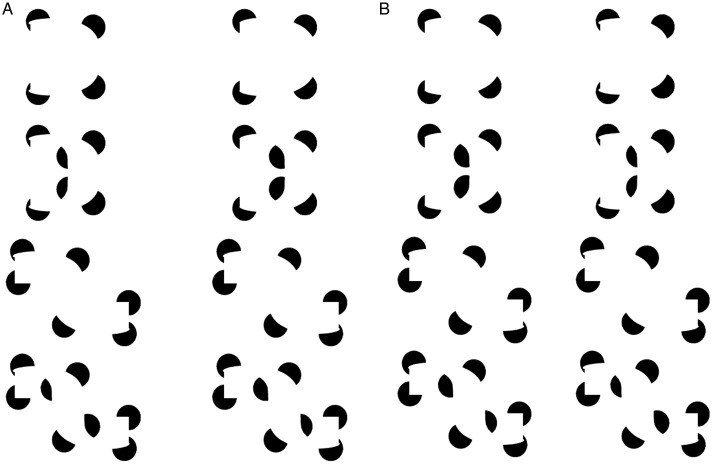
Adding semioccluded balls transforms the closed surfaces of (a), shown in the top row, or (c), into the open surfaces of (b) or (d): a crossed-disparity version (A) and an uncrossed-disparity version (B).

In Figures 2a and 2c, closed surfaces are perceived. That is, volumes are perceived whose rim is defined by the outer contours of the object, and whose interpolated modally completing surface completes in a smooth or analog manner across the visible portion of the object. However, adding intervening semioccluded “balls” as in Figure 2b and 2d converts these closed surfaces, or volumes, into open surfaces that wrap around the balls. This leads to an atypical type of modal completion in Figure 2c: In Figure 2c, visible surface fragments complete with visible surface fragments, whereas in Figure 2d, visible surface fragments now complete with *non*-visible surface fragments, that are occluded by that own surface’s back side, as we saw in Figure 1d.

**Figure 3.**
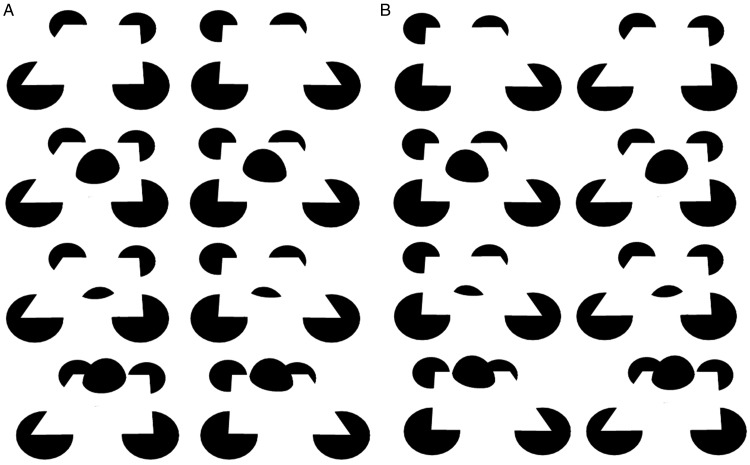
Embedding an “egg” in a flat modally completing surface (a, top) can lead it to instead appear pushed down, as in (b) or (c), or pulled up, as in (d): a crossed-disparity version (A) and an uncrossed-disparity version (B).

In Figure 3a, a rather standard slanted square sheet completes in the style of Kanizsa. However, embedding an “egg”^1^ as in Figure 3b and 3c bends the modally completing surface smoothly rather like pressing down into a rubber sheet or like common drawings that render the curvature of space–time around a celestial mass. In contrast, in Figure 3d, raising the same embedded object above that surface does not lead to the impression that the “egg” is pressing into a rubber sheet, but instead leads the modally completing surface to be pulled up toward that object, again in a manner that preserves analog smoothness or differentiability. In the past, I have argued (Tse, 1998) that objects tend to conform to their supporting surfaces, such as the ground plane, in light of an implicit assumption of surface attachment (Albert & Tse, 2000). But here, the supporting surface adheres to or conforms to the egg, as if it were a rubber sheet attached to the embedded egg and “pulled up” by it. This might follow from the implicit assumption of surface attachment because the alternate interpretation would be that a nonoccluded hemiegg is floating in the air. But this is an unlikely scenario because the contour curvature discontinuities of the lower portion of the egg in the image are strong cues that it is embedded in an occluder, volumetrically (Albert & Tse, 2000; Tse, 1998).

**Figure 4.**
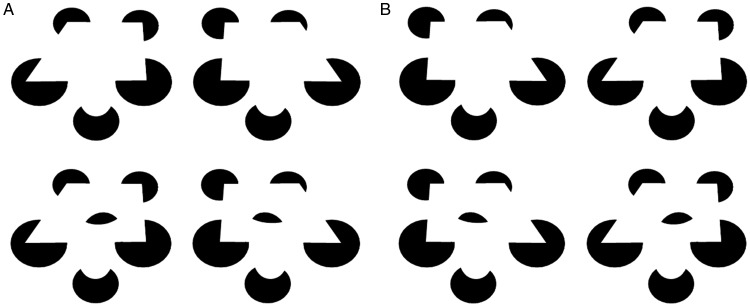
Here the underside of the “pressed-down surface” of Figure 3b and c has been made visible: a crossed-disparity version (A), an uncrossed-disparity version (B), an animated crossed-disparity version (Movie 3), and an uncrossed-disparity version (Movie 4).

In Figures 3b and 3c, the egg presses down into the surface, but the underside of the embedding surface is not visible. In Figures 4a and 4b, the surface has been made explicit as a “cone.” Whereas in Figure 4a, its relationship to the square surface above it is ambiguous, in Figure 4b, it unambiguously completes as the underside of the surface pressed down by the egg. In the animated version shown in Movies 3 and 4, the underside “cone” appears to unambiguously complete with the square surface when there is an egg growing or pressing down into it but only ambiguously appears to link up with the square surface in the absence of the moving egg.

**Movie 3.** (click to play). Crossed Disparity.

**Movie 4.** (click to play). Uncrossed Disparity.

**Movie 5.** (click to play). Crossed Disparity.

**Movie 6.** (click to play). Uncrossed Disparity.

**Figure 5.**
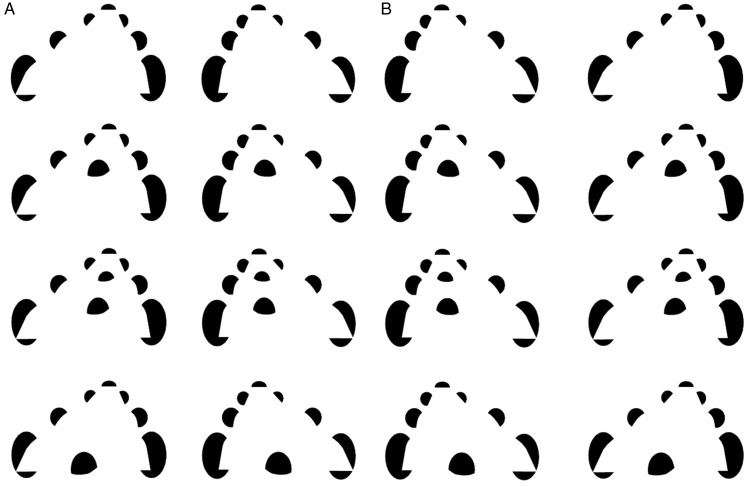
Here the wavy slide shown in (a, top) can be pressed down in various interesting ways by eggs in (b), (c), and (d): a crossed-disparity version (A), an uncrossed-disparity version (B), an animated crossed-disparity version (Movie 5), and an uncrossed-disparity version (Movie 6).

Building on this, the curved open surface of Figure 5a is rather like a wavy slide. However, embedding a shape, as in Figures 5b to 5d, warps the shape of the slide, as in Figures 3b or 3c, such that it warps around the embedded object. An animated version of this is shown in Movies 5 and 6.

**Figure 6.**
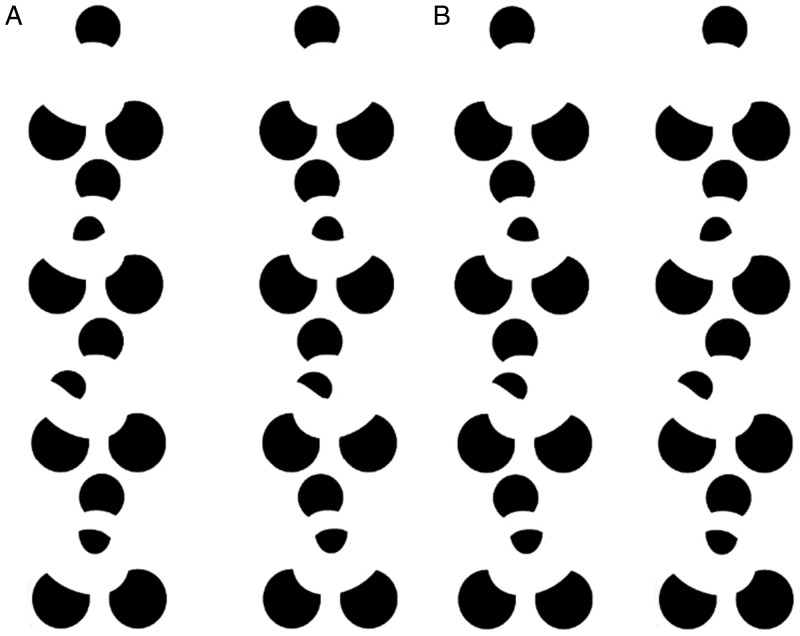
Embedding an “egg” in a curved modally completing surface (a) can lead it to transform from a “pringle” to a volumetric “nest” (b), “tongue” (c), or “bell” (d): a crossed-disparity version (A) and an uncrossed-disparity version (B).

In Figure 6a, the perceived shape is an open surface that is saddle-shaped, rather like a pringles potato chip. Thus, contours are taken to arise from edge here, not rim. However, adding an embedded “egg” as in Figure 6b transforms the perceived surface into a closed surface or volume in the shape of a 3D “nest.” Now the same bounding contours in the image are taken to arise from rim, not edge. Moving the egg can radically change the 3D structure of the perceived volume, as in Figure 6c. And flipping just the “egg” portion of Figure 6b upside down, as in Figure 6d, surprisingly transforms the “nest” that is perceived when viewing Figure 6b, into a 3D “bell,” seen from below, even though the bounding contours are the same in all figures of Figure 6. Again, an image contour that was taken to arise from edge in Figure 6a, now appears to arise from rim, at least at the top of the bell. The bottom of the bell arguably arises from edge.

## Discussion

The main point of these demonstrations is that surface and volume completion cannot be solved in terms of local contour linkages or local surface interpolations, or a succession or stack of such localistic interpolations. Rather, the entire scene, with its relationships of parts to other parts over potentially tens of visual degrees, must be considered as a whole. What is remarkable is that a local change in the “abutting” object can flip the interpretation of the cause of a contour in the image that is far away from that object, for example, flipping rim into edge or flipping a convex surface into a convex one, as in Figures 6b and 6d.

Local interpolations are a consequence of global interpolations, rather than the other way around. How the visual system executes such global interpolations is an open question. Indeed, the fields of Psychology and Neuroscience have so far been unable to fully rise to the challenge posed by the Gestalt Psychologists a century ago concerning the nature of grouping operations across the visual field that go into the construction of consciously experienced vision. This may be in part because of a reductionistic bias and agenda in our field, according to which everything can be reduced to the tuning properties of localized receptive fields, or transformations of such detected information through a bottom-up stack of local operations. In such a reductionistic and localistic picture, where information is detected and then processed, there is little room for the addition of information not even implicitly in the image or its creation. For example, according to the now dominant local-prior-to-global view, features are detected locally. But what is a feature? If what happens tens of visual degrees away can lead to local motion being perceived to be either leftward or its opposite, rightward (Tse, 2006, Figure 2), how can we argue that leftward or rightward motion is detected locally and independently of distant inputs? We cannot. Similarly, in the domain of 3D shape considered here, if the 3D surface slant attributed to a contour in the image can change radically depending on distant inputs, as we see in Figure 6, can we argue that surface features are detected locally and independently of distant inputs? Again, I believe we cannot. The same points could be made for color, brightness, and other so-called primitive features. Therefore, the local-to-global worldview is incomplete and must be integrated with a global-to-local perspective according to which local features are determined only after inputs are compared and analyzed over space and time. This does not mean that we have to get rid of the useful idea of a neural receptive field. Surely a V1 neuron, for example, responds to a limited range of inputs in terms of both spatial extent and image properties. Such properties are not the same as the features that we experience in visual consciousness, such as redness, brightness, direction of motion, slantedness, position in 3D, and so forth. Such consciously experienced features are derived from what has been detected at early stages but are not the same as what has been detected at those stages.

An alternative view is that vision is very much like hallucination, but one that has evolved to be as veridical as possible, so we can function in the real world. Of course, even a veridical hallucination must begin with what has been detected at the receptor level, but vision is more fundamentally about what is constructed on the basis of what has been detected. Just because what is detected locally by photoreceptors can be thought to be detected independently of what may be detected nonlocally, by other receptors, does not mean that the constructive processes that create conscious perception must operate localistically, in space or time. Indeed, these and other demonstrations that I and others have made over the decades drive home the holistic nature of constructive processes underlying vision and other aspects of experience.

Another point made by these demonstrations is that completion cannot easily be broken down into facile categories such as surface versus volume completion or modal versus amodal completion. For example, the front visible surface fragments in Figure 1d appear to complete with a nonvisible surface, whose back side is visible as the “basin,” which occludes its own front side. The same goes for Figure 2d. Completion therefore need not involve the linkage of two or more *visible* surface fragments. Indeed, if modal completion links together visible and nonvisible, occluded surfaces, the distinction between modal completion (i.e., in front, such that visible fragments link to visible fragments via interpolated visible surfaces) and amodal completion (i.e., behind an occluder, such that visible surface fragments link to visible fragments via interpolated nonvisible surfaces) is a distinction that is no longer particularly useful (see also Scherzer & Faul, 2019); instead, we should simply talk about surface and volume completion or interpolation in 3D.

As discussed first in Tse (2017a, 2017b), but now repeated here, several ideas have been put forth regarding surface interpolation processes in both the psychological and computer vision fields. Some surface completion algorithms (Sarti et al., 2000) have been able to account for the flat surface perceived in the Kanizsa triangle by viewing the problem as one of minimizing surface curvature of a Riemannian manifold whose metric properties are constrained to meet conditions imposed by image contours. The first step in almost all such algorithms is to detect image boundaries. This is followed by a step of surface evolution that is attracted to edges, and which also completes missing edges and surfaces among and between visible edges according to a surface smoothness constraint that follows from the minimization of Riemannian curvature, as would occur for a soap bubble suspended between wires at the locations and depths of the image contours.

But in the cases considered here and in Tse (2017a, 2017b), the surfaces interpolated by the visual system behave very differently than soap bubbles hanging among wires defined by visible contours. Unlike soap bubbles, surfaces here can appear to pass through visible contours along the line of sight tangentially as would occur when looking at a smooth closed surface (e.g., a potato), rather than orthogonally to it, as in the traditional Kanizsa triangle case. That is, in the cases demonstrated here or in Tse (2017a, 2017b), contours are not taken to arise from 3D edges, where surfaces end; instead, they are taken to arise from 3D rim, where the line of sight tangentially grazes a smooth surface that continues smoothly away from the rim, in both forward (visible, toward the viewer) and backward (nonvisible, away from the viewer) directions.

Rather than propagate surfaces toward visible contours, other algorithms propagate curved surfaces inward from visible contours. At least one such contour curvature propagation algorithm (Tse, 2002) depends on the availability of apparent planar cuts along a visible contour, specified as segments of contour between contour curvature discontinuities, which can then reveal information about the 3D cross-section of a volume. But the disks used to create the demonstrations in Tse (2017a, 2017b) did not carry any such planar cut information in their contours, yet nonetheless appeared volumetric. The same goes for some of the demonstrations here, so more must be going on than contour propagation, or inferred cross-section propagation from the visible contour inward to regions lacking depth information.

An alternative idea is the idea of an attentional shroud (Cavanagh et al., 2001; Fazl et al., 2009; Moore & Fulton, 2005; Tyler & Kontsevich, 1995) that places a mesh among visible contours, and which can have a certain rigidity among nodes of the mesh, limiting it from collapsing into a soap bubble solution. But rigidity of a default surface mesh cannot easily account for the fact that sometimes interpolated surfaces pass through visible contours along the line of sight (rim), and other times are interpolated to lie orthogonal to the line of sight (edge), depending on contour information present far away in the image. Future theoretical work will have to explain why 3D open surfaces are interpolated for some of the cases shown here, for example, in Figure 6a, but why closed surfaces or volumes are interpolated for other figures here, as in Figure 6b. The bounding contours are the same in both Figures 6a and b, and yet one case is interpolated to arise from edge (of a pringle potato chip), while in the other case, the exact same bounding contour is taken to arise from rim (of a nest). Thus, one question that the present demonstrations raise is: “How does the visual system decide that a detected contour should be constructed to have arisen from edge or rim in the world?” Clearly, a simple, complex, or hypercomplex cell in V1, facing as it does an aperture problem, cannot alone solve this problem. But then, at what level of neural processing, do neuronal responses distinguish between different world solutions (e.g., rim vs. edge) that are consistent with image cues?

While speculative, the idea of an attentional shroud or 3D encompassing surface or manifold could in principle be realized in something like the “grid cells” (Moser et al., 2014) known to specify a coordinate system for a 3D layout. However, to date, no one has found or even proposed analogous “mesh cells” for 3D objects or surface representations of objects. If such cells exist, which would lay down a 3D mesh within visible contours of objects, in a manner analogous to the laying down of a grid by grid cells within visible borders, a possible place to look for them might be among recently described (Yau et al., 2013) 3D surface curvature cells in visual area V4. At this point, however, the existence of such cells is purely speculative.

Certain models have made explicit that surfaces can be completed from visible contour fragments within and between depths. Notable among these is the Boundary Contour System/Feature Contour System model of Grossberg and Mingolla (1985 a,b) and the later elaboration of that theory called “FACADE theory” (Grossberg, 1994). These models posit “bipole cells” that complete contours that are adequately coaligned based on good contour continuation in the image. If that level of completion fails, then the second stage of surface “diffusion” away from the completed contour does not take place. And even if it begins, diffusion can get blocked by a visible boundary (Mumford & Shah, 1989; Neumann et al., 1998; Perona & Malik, 1990; Proesmans & Van Gool, 1999). Problems arise for such models in that contour continuity can occur in the image that arises when two occluded volumes are in fact not connected in the world. Contour continuity can occur, but surface completion fails for other reasons. Indeed, a volume can complete even when there are no coaligning contours in the image at all (Tse, 1999a, 1999b); thus, contour continuity in the image is neither necessary nor sufficient for amodal or modal completion. Moreover, relying on contour relatability alone cannot explain the different perceived surfaces in Figures 3 or 6. More must be going on than connecting contour fragments and then filling in surfaces within completed contours.

More recently, some authors (e.g., Kogo et al., 2010) have emphasized that depth ordering is the primary problem that must be solved, followed by surface completion within the contours at a given depth. But this approach still cannot account for surfaces that complete smoothly behind, between, and even in front of the depth planes explicitly given by image contour disparity cues.

Whether any local portion of image contour is interpreted to arise from a surface edge or surface rim cannot be decided based solely on local cues. The rim interpretation, where the line of sight is taken to tangentially graze a differentiable surface, requires that a distal portion of rim exists to which the surface can link as a smooth manifold that can bend as much as 180 degrees in space. But if one counts the self-occluded regions of the closed surfaces perceived in many cases considered here, the surface inferred to pass through the rim must bend through 360° in order to close in on itself from the far, self-occluded side.

In conclusion, the present demonstrations extend and go beyond the insights about visual processing raised by Gaetano Kanizsa’s (1955) triangle figure or the open curved surfaces introduced by Carman and Welch (1992), or my own past work (Tse, 2017a, 2017b). Unlike those classes of examples, the present demonstrations make plain the extent to which surface interpolation is a nonlocalistic 3D completion process, not limited to the flat or curved open surfaces. In the present demonstrations, smooth surfaces are interpolated to span two or more discrete disparity-defined depths and often close on themselves to form a volume. Such interpolated surfaces are quite radically altered by the addition of an abutting object far from the “pacmen” traditionally associated with generating cues to modal and amodal completion. That two or more discrete contour fragments at discrete disparities can give rise to an analog representation of a unified open or closed 3D surface that is remarkable. And that this surface is globally warped by the addition of a small object assumed to be abutting that surface is even more remarkable. This nonlocality is a testament to the daily creativity of the constructive holistic processes that underlie everyday 3D vision (see Koenderink, 2015, for more on this theme).

No existing theoretical or computer models of visual processing can fully account for the 3D surfaces and volumes perceived in the present demonstrations or those of Tse (2017a, 2017b). It can only be hoped that future modeling and neurophysiological research will be able to explain why these 3D surfaces are perceived rather than the many others that are also consistent with the present image cues. There are still deep unknowns concerning surface completion; for example, why is an edge solution sometimes preferred over a rim solution? This clearly cannot be a localistic solution because an identical image contour is interpreted to be edge in Figure 6a but rim in Figures 6b to d. But if a modally completing surface must satisfy global constraints, how is this done? Again, the challenge first posed by the Gestalt Psychologists in this regard has yet to be fully met. Yes, perception is a construction, even a veritable “veridical hallucination” based upon what was detected by sensory neurons. But how is visual perception constructed? Despite much progress in Perceptual Psychology and Neuroscience, we are still a field far from completion.

## Supplementary Material

Supplementary material

## Note

1 I chose to use an “egg” because it generally lacks image tangent discontinuities, even if embedded, as proven in Tse and Albert (1998). However, the presence of contour curvature discontinuities in the image provides a cue to the presence of surface discontinuities in the world (Kristjansson & Tse, 2001; Tse, 2002). In the case where the egg seems to be pushed down into the surface, however, the contour tangent discontinuities are cues that the embedded egg is occluded by the underlying surface at a distance, rather than wrapped around the egg without a gap, as would occur if an egg were placed half in water. This “at a distance” occlusion is what leads to the impression that most of the egg lies inside a curved, funnel-shaped depression.
