# Supplementary figures and images for: Abutting Objects Warp the Three-Dimensional Curvature of Modally Completing Surfaces
Source: Iperception. 2020 Apr 9;11(2):2041669520903554. doi: 10.1177/2041669520903554 (PMC7253068; doi:10.1177/2041669520903554)

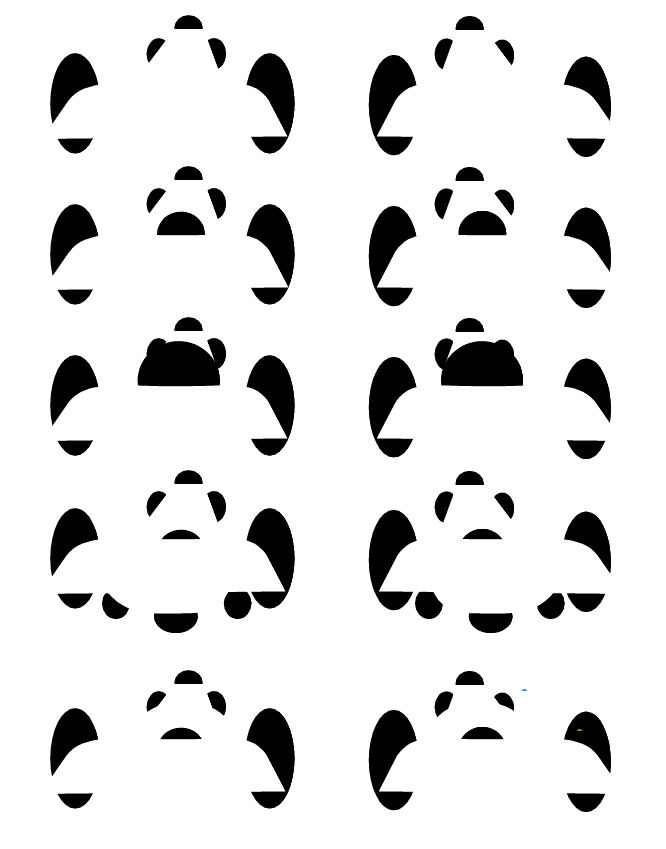

Supplement: Supplementary material [file IPE903554_Supplemental_Material.zip › Figure1crossed.jpg]

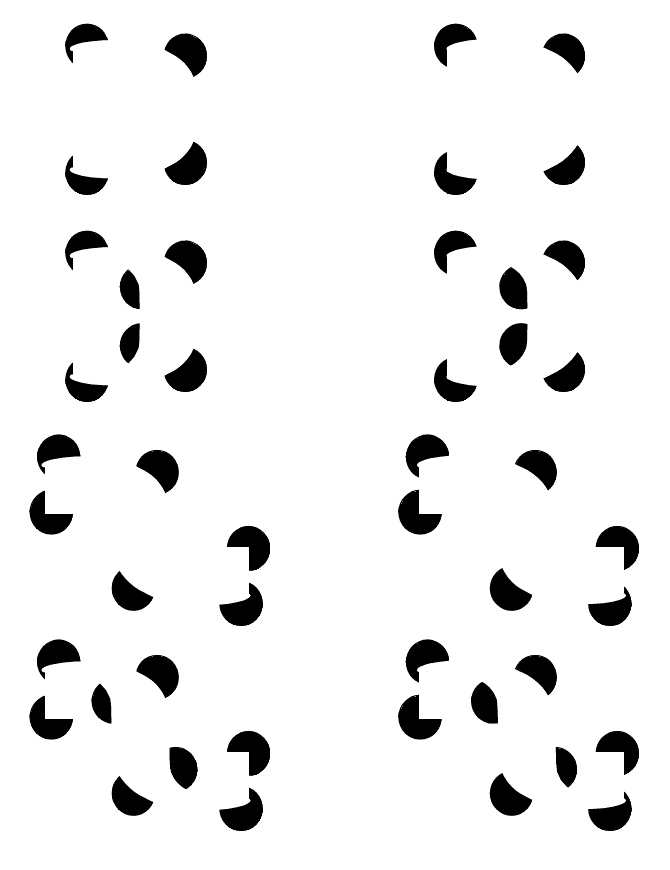

Supplement: Supplementary material [file IPE903554_Supplemental_Material.zip › Figure2crossed.jpg]

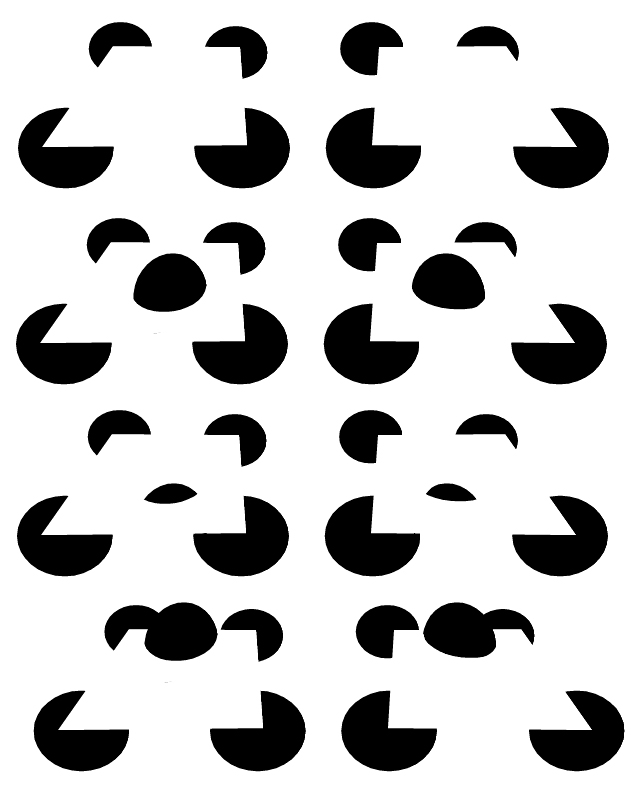

Supplement: Supplementary material [file IPE903554_Supplemental_Material.zip › Figure3crossed.jpg]

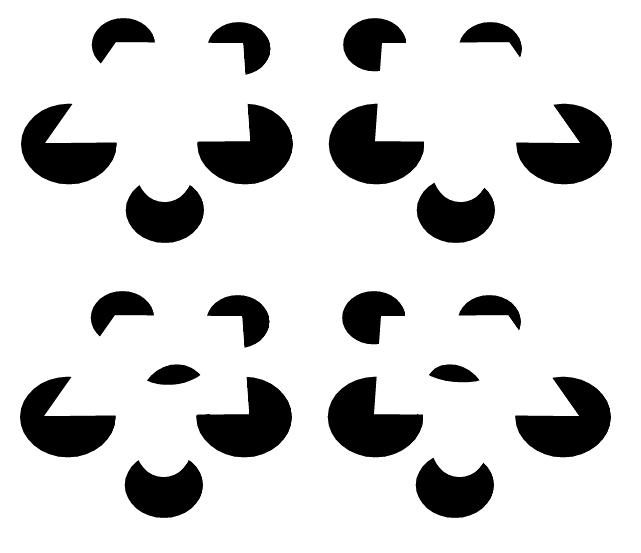

Supplement: Supplementary material [file IPE903554_Supplemental_Material.zip › Figure4crossed.jpg]

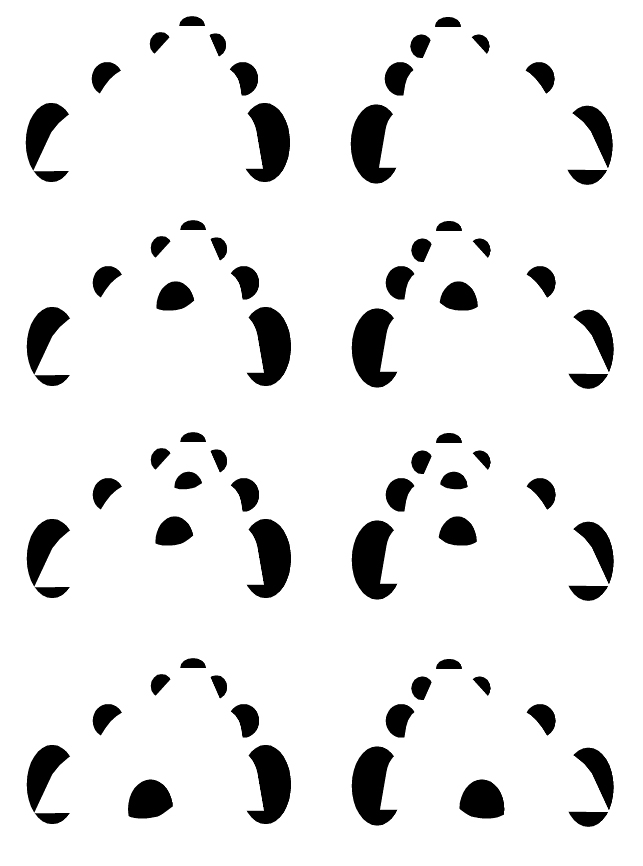

Supplement: Supplementary material [file IPE903554_Supplemental_Material.zip › Figure5crossed.jpg]

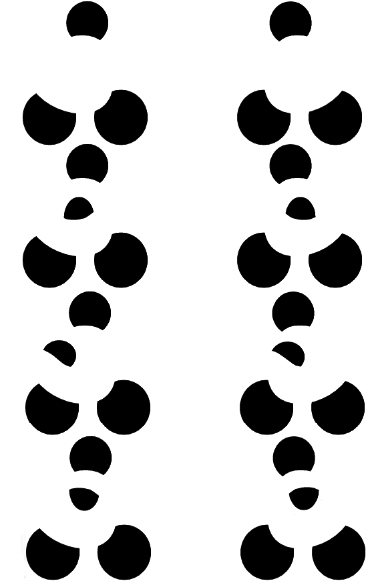

Supplement: Supplementary material [file IPE903554_Supplemental_Material.zip › Figure6crossed.jpg]

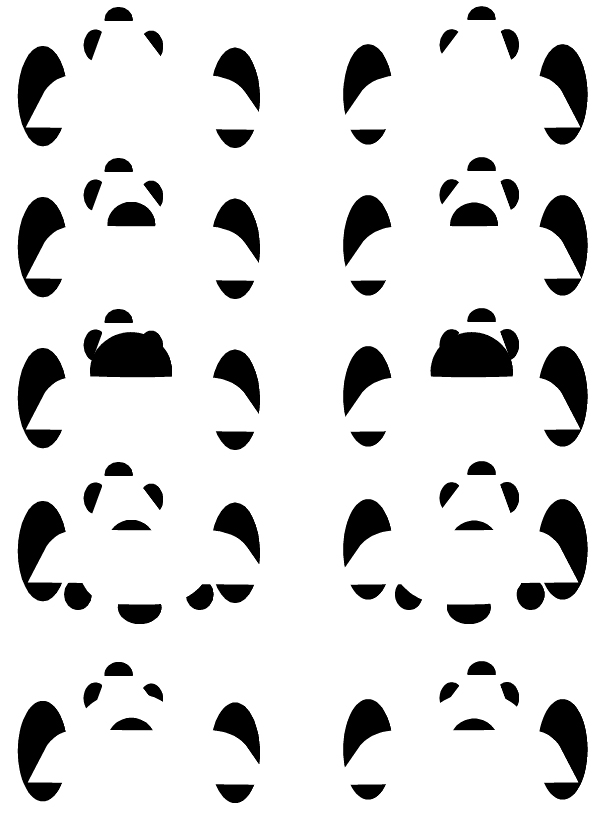

Supplement: Supplementary material [file IPE903554_Supplemental_Material.zip › Figure1uncrossed.jpg]

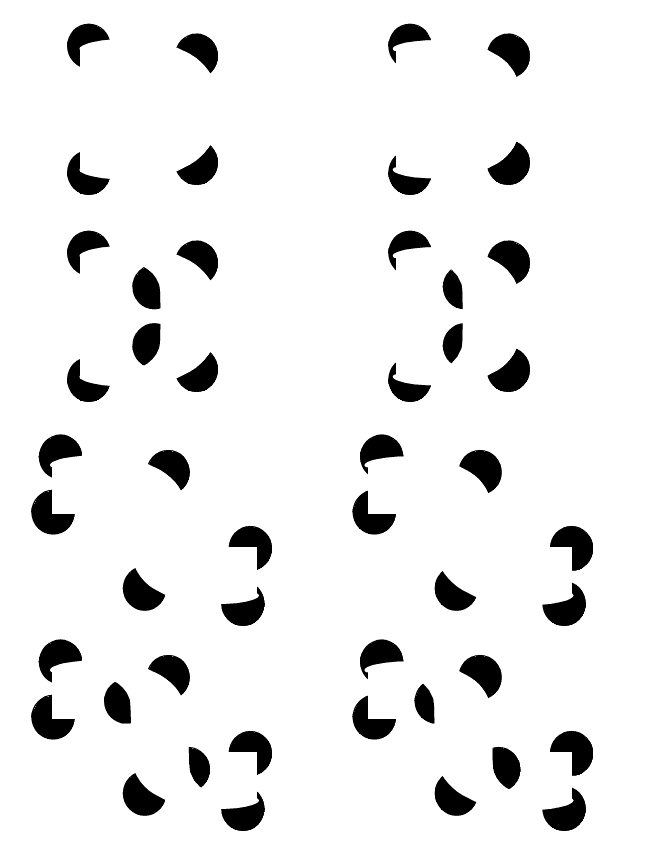

Supplement: Supplementary material [file IPE903554_Supplemental_Material.zip › Figure2uncrossed.jpg]

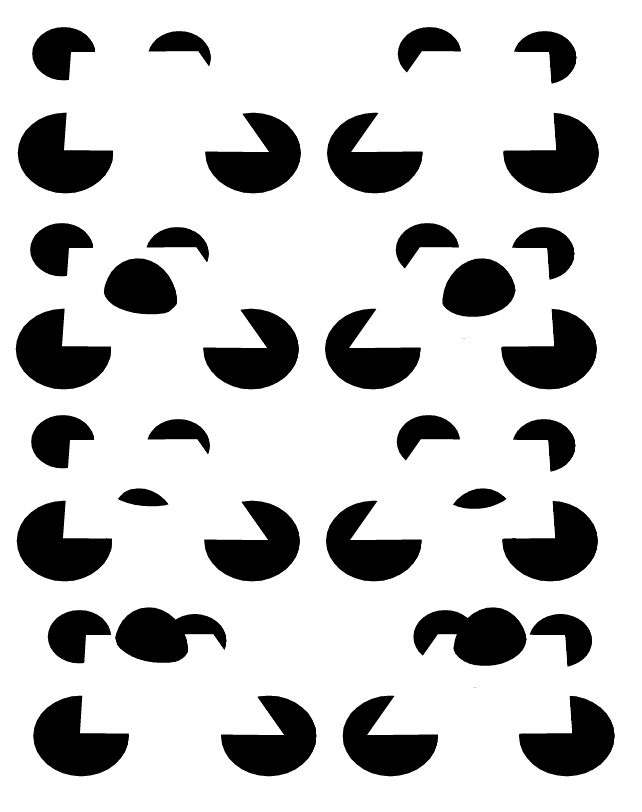

Supplement: Supplementary material [file IPE903554_Supplemental_Material.zip › Figure3uncrossed.jpg]

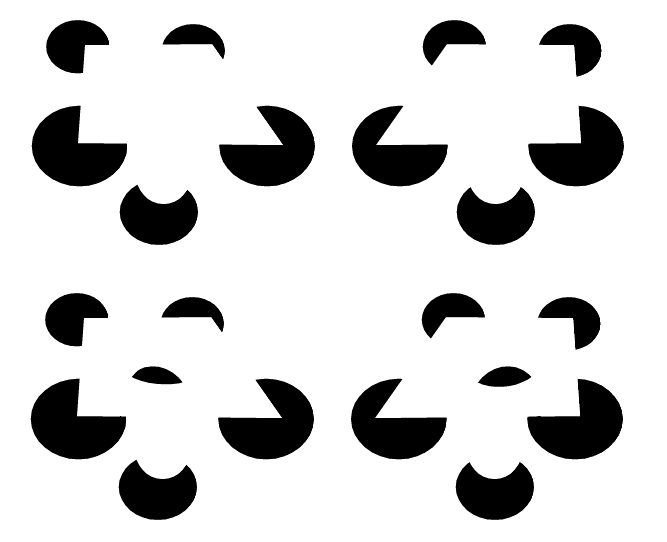

Supplement: Supplementary material [file IPE903554_Supplemental_Material.zip › Figure4uncrossed.jpg]

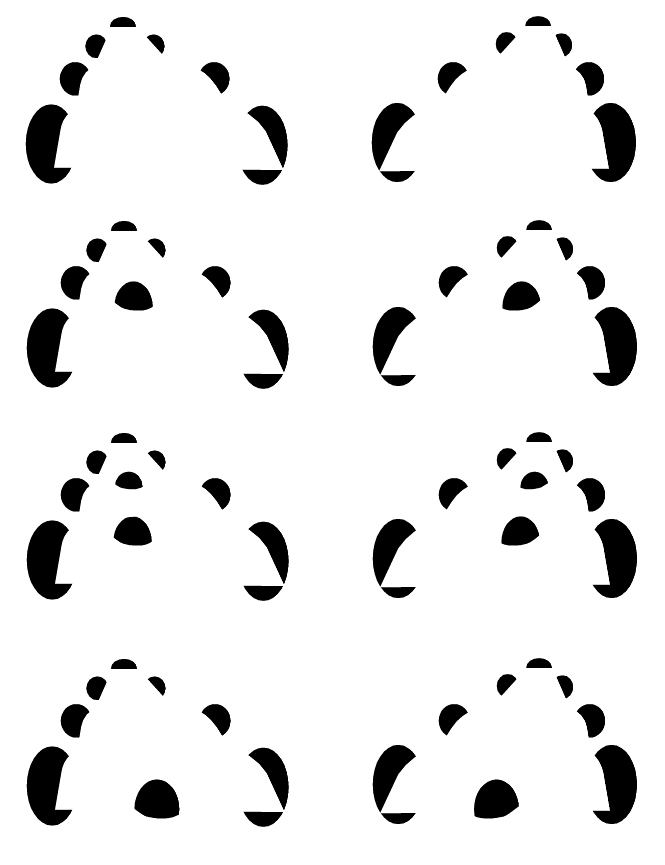

Supplement: Supplementary material [file IPE903554_Supplemental_Material.zip › Figure5uncrossed.jpg]

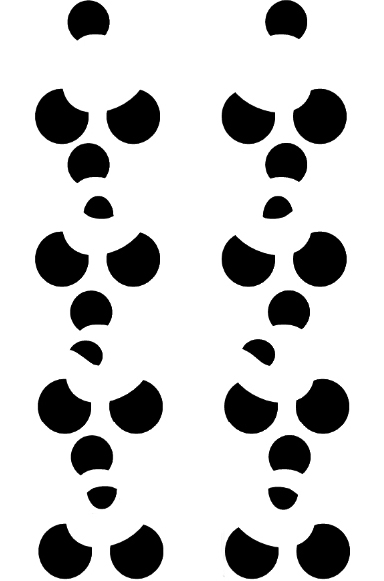

Supplement: Supplementary material [file IPE903554_Supplemental_Material.zip › Figure6uncrossed.jpg]

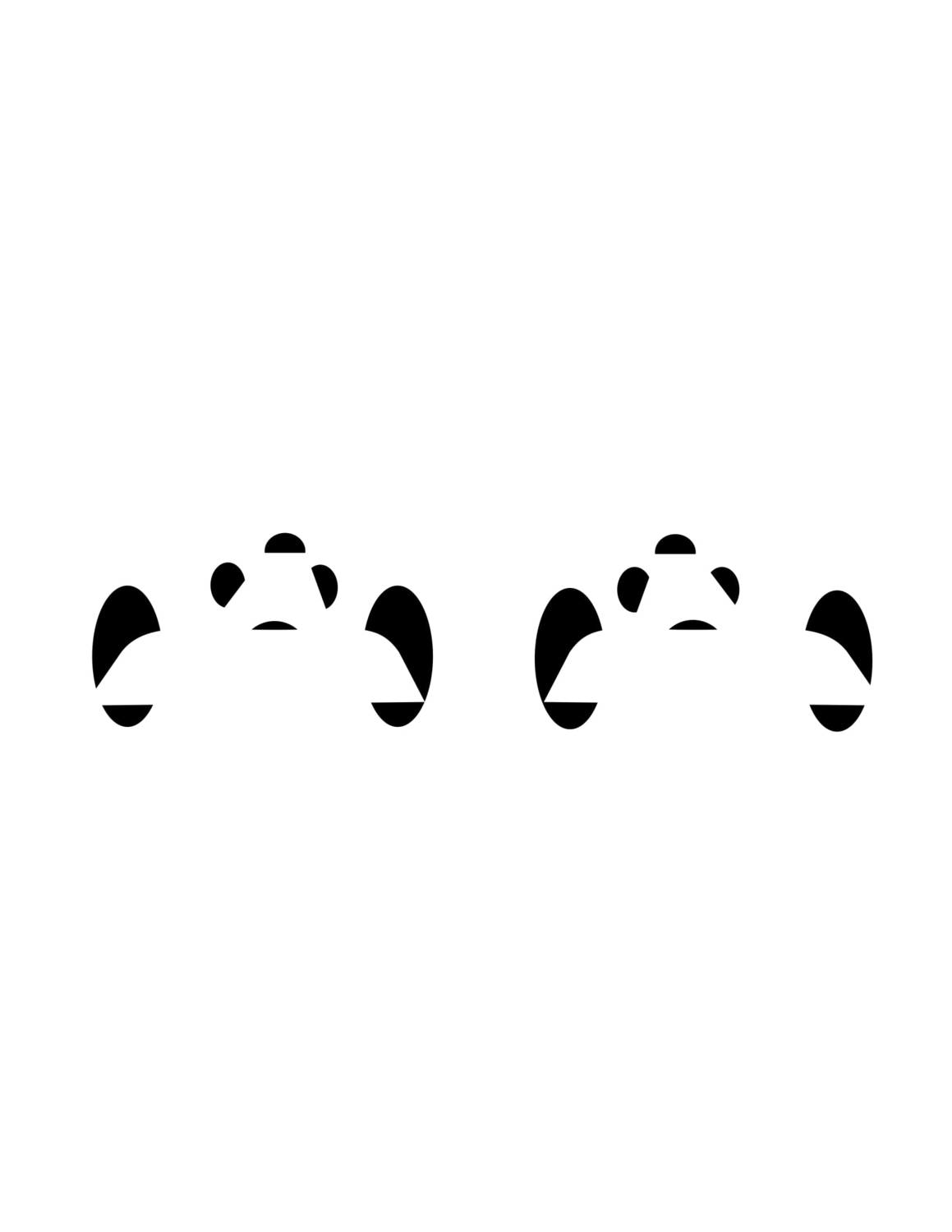

Supplement: Supplementary material [file IPE903554_Supplemental_Material.zip › Figure1Animation1crossed.gif]

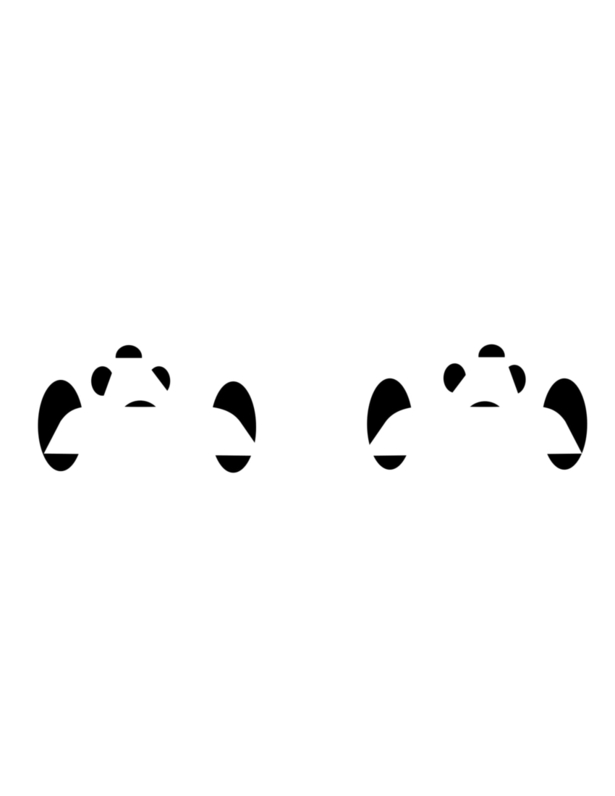

Supplement: Supplementary material [file IPE903554_Supplemental_Material.zip › Figure1animation1uncrossed.gif]

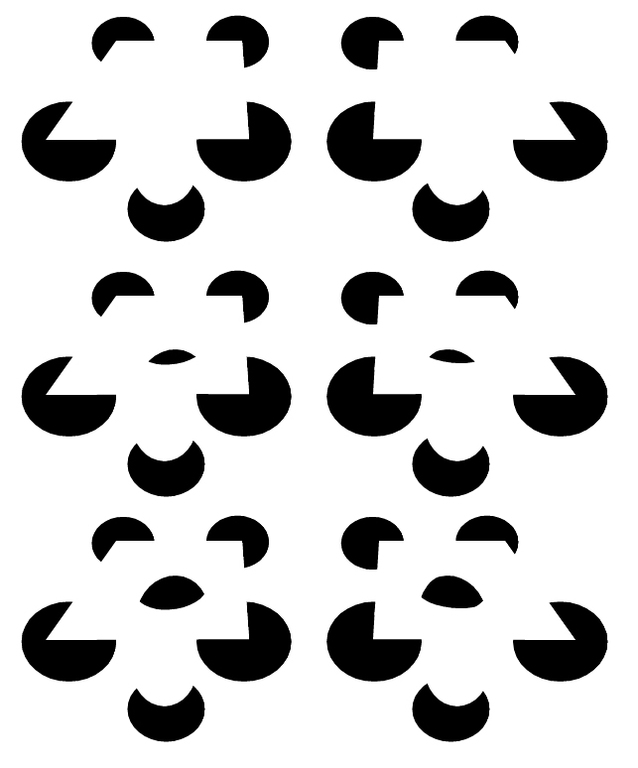

Supplement: Supplementary material [file IPE903554_Supplemental_Material.zip › Figure4Animation2crossed.gif]

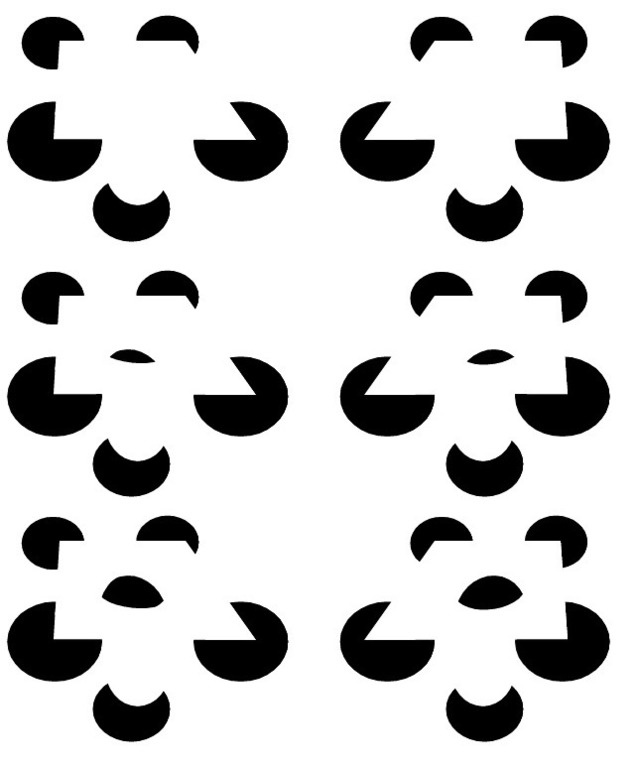

Supplement: Supplementary material [file IPE903554_Supplemental_Material.zip › Figure4Animation2uncrossed.gif]

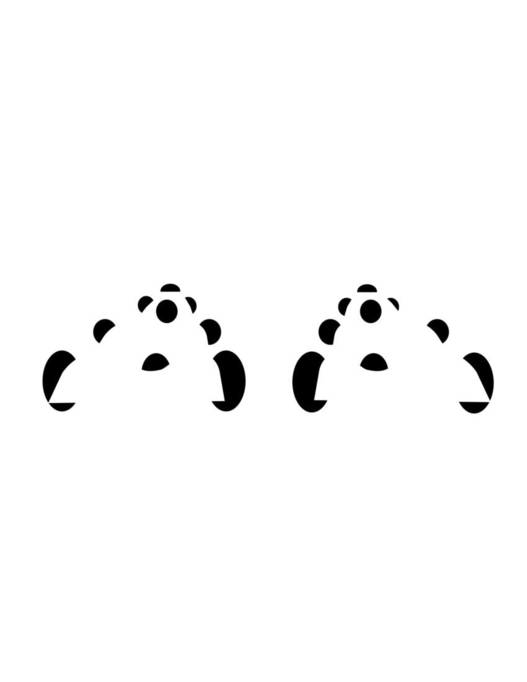

Supplement: Supplementary material [file IPE903554_Supplemental_Material.zip › Figure5Animation3crossedv.gif]

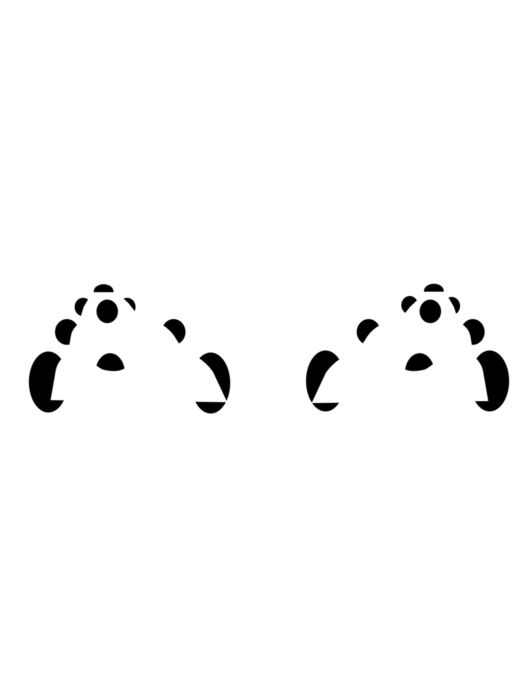

Supplement: Supplementary material [file IPE903554_Supplemental_Material.zip › Figure5animation3uncrossed.gif]
